# Deconvolution of bulk RNA sequencing in activated phosphoinositide 3‐kinase δ syndrome

**DOI:** 10.1111/crj.13702

**Published:** 2023-09-27

**Authors:** Xia Huang, Haiyan Gu

**Affiliations:** ^1^ Department of Respiratory Medicine Children's Hospital of Nanjing Medical University Nanjing China

**Keywords:** CIBERSORT, PI3Kδ, primary immunodeficiency, RNA‐seq

## Abstract

**Background:**

Many gaps remain in our understanding of the immune and molecular characteristics that underlie activated phosphoinositide 3‐kinase delta syndrome (APDS).

**Methods:**

We performed RNA sequencing of peripheral blood leukocytes obtained from a child with APDS and his healthy parents and deconvoluted bulk transcriptional data to assess immune cell status.

**Results:**

Pathway enrichment analysis suggested signaling pathways enriched in virus infection as well as the PI3K, mitogen‐activated protein kinase (MAPK), natural killer cell‐mediated cytotoxicity, and nucleotide‐binding oligomerization domain (NOD)‐like receptor signaling pathways. The proportion of B cells memory, T cells CD4 memory resting and dendritic cells activated were reduced, whereas B cells naïve, T cells CD8, NK cells resting, monocytes and macrophages M2 were increased in the child. Top 10 hub genes were screened and showed moderate to strong relatedness with immune cell proportions.

**Conclusion:**

Deconvolution of bulk RNA sequencing to assess immune cells status can provide further insight into the alterations in immunological features underlying APDS and other rare diseases.

## Graphical abstract

Pathway enrichment analysis suggested signaling pathways enriched in virus infection as well as the PI3K, mitogen‐activated protein kinase (MAPK), natural killer cell‐mediated cytotoxicity, and nucleotide‐binding oligomerization domain (NOD)‐like receptor signaling pathways. Eight differentially altered immune cells were detected in APDS. All the 10 hub genes showed moderate to strong relatedness with immune cell proportions.

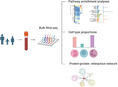

Activated phosphoinositide 3‐kinase delta syndrome (APDS) is a rare primary immunodeficiency disorder characterized by a spectrum of clinical manifestations, including recurrent respiratory tract infections, lymphoproliferation, hyper‐immunoglobulin (Ig) M, and low Ig G levels in the serum and impaired vaccine responses.^1^ APDS mostly results from gain‐of‐function mutations in the *PIK3CD* gene, which encodes the catalytic p110δ subunit of phosphoinositide 3‐kinase δ (PI3Kδ), leading to hyperactivation of PI3Kδ and downstream Akt signaling with a consequent effect on immune cell development and differentiation.^2^, ^3^ However, phosphoinositide 3‐kinase (PI3K)/Akt pathway‐targeted therapies such as rapamycin have limited effects in certain populations.^3^ Therefore, many gaps remain in our understanding of the immune and molecular characteristics that underlie APDS. In the present study, we performed RNA sequencing of peripheral blood leukocytes obtained from a child with APDS and his healthy parents and deconvoluted bulk transcriptional data to assess their immune cell status, including alterations in immunological features, immune function, and signaling pathways caused by continuous PI3K pathway activation.

The 4‐year‐old boy experienced recurrent wheezing, pulmonary infection, airway stenosis, non‐neoplastic lymphoproliferation, herpesvirus infection, increased serum IgM levels, and serum IgG deficiency. He was diagnosed with APDS by whole‐exome sequencing in the proband and parents, which revealed a heterozygous mutation in *PIK3CD* (c.3061G > A [E1021K]) on chromosome 1. The child's clinical features were detailed in Figure 1. Detailed materials and methods were described in Data S1.

**FIGURE 1:**
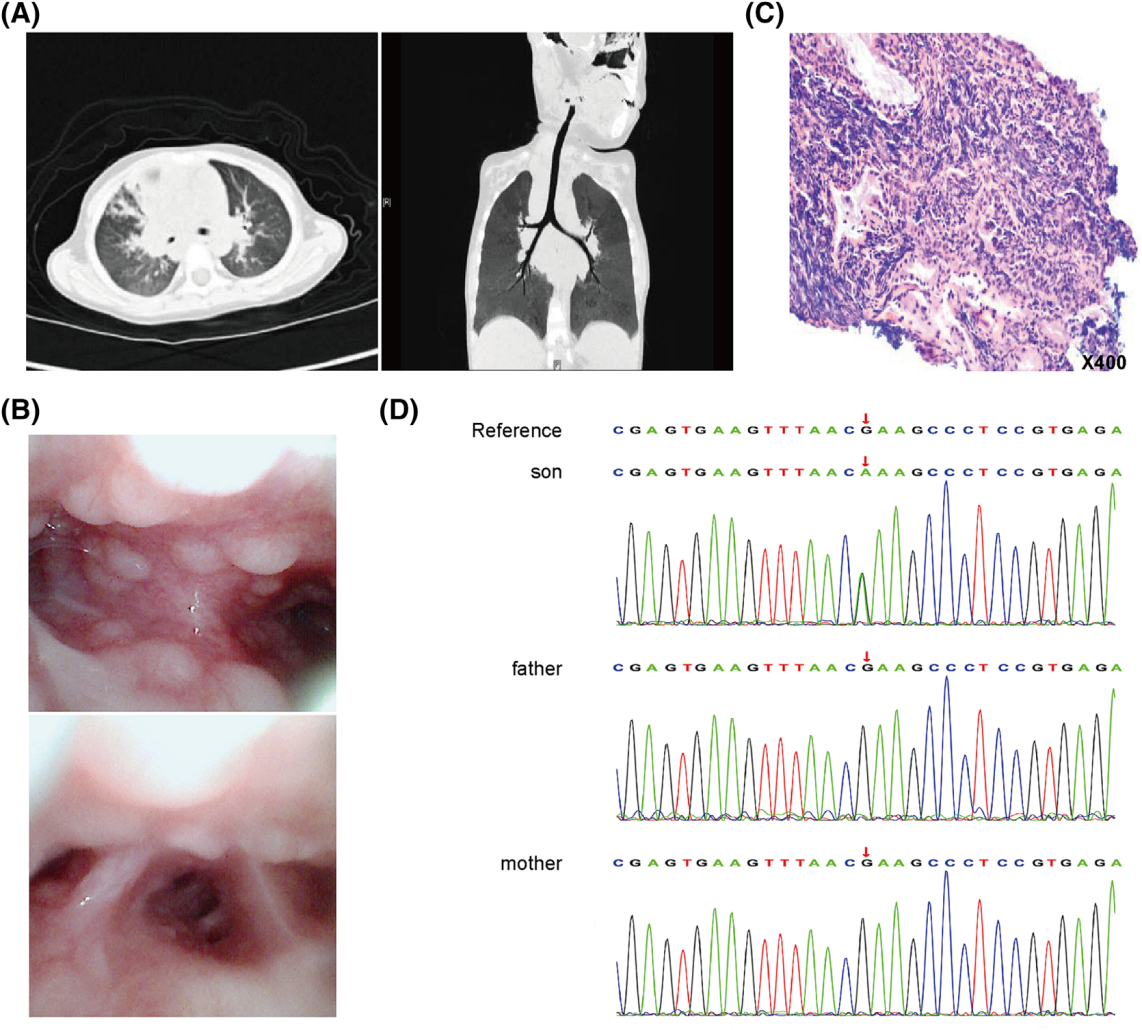
Clinical features of a 4‐year‐old boy with activated phosphoinositide 3‐kinase delta syndrome (APDS). (A) Chest computed tomography image revealing bilateral pneumonia, proximal bronchial stenosis in the middle segment of the right lung, and widening of the right upper mediastinum. (B) Bronchoscopy image revealing follicular hyperplasia of the trachea and bronchial mucosa. (C) Histopathology image of the bronchial mucosa indicating lymphocyte infiltration and accumulation in the bronchial mucosa (hematoxylin–eosin staining). (D) Family sequencing showed a heterozygous mutation in the *PIK3CD* gene (c.3061[exon24] G > A) on chromosome 1 in the child, and neither parent had the mutation.

A total of 1282 differentially expressed genes (DEGs) were identified between the son and mother (815 upregulated, 467 downregulated), while 2053 DEGs were identified between the son and father (1447 upregulated, 606 downregulated). The overall expression of DEGs between the child and his parents were displayed in the heat maps (Figure 2A). The DEGs were then subjected to both Gene Ontology (GO) function and Kyoto Encyclopedia of Genes and Genomes (KEGG) pathway enrichment analyses to predict their potential biological functions. The most significant enrichment terms for GO were immune system process, adaptive immune response, innate immune response (biological process), membrane plasma membrane and nucleus (cellular component), protein binding, metal ion binding, and nucleotide binding (molecular function) (Figure 2B). The DEG signaling pathways were mainly enriched in virus infection as well as the PI3K, mitogen‐activated protein kinase (MAPK), natural killer cell‐mediated cytotoxicity, and nucleotide‐binding oligomerization domain (NOD)‐like receptor signaling pathways (Figure 2C and D).

**FIGURE 2:**
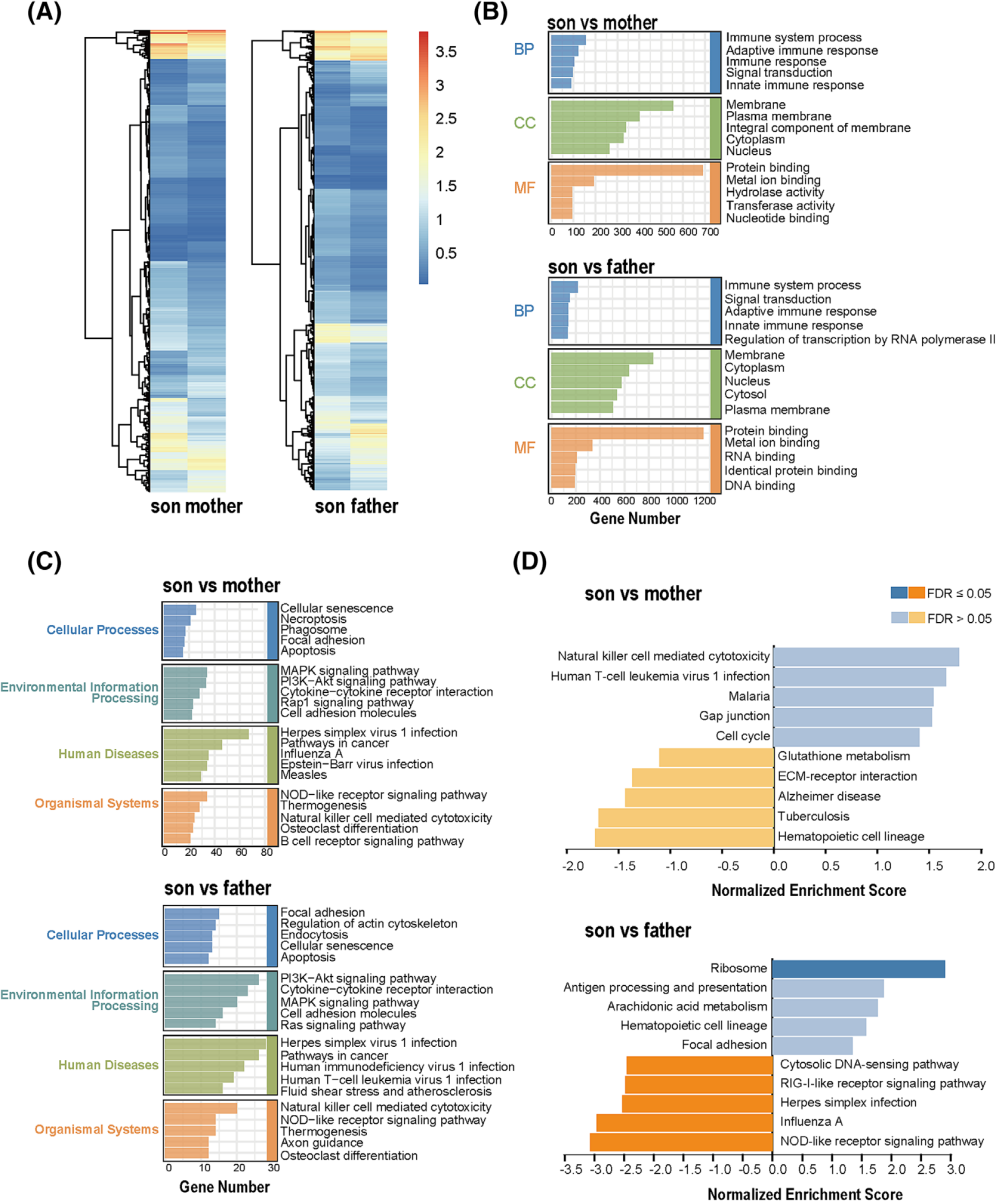
DEG enrichment analysis. (A) Heatmaps showing the DEGs. The gradation of color represents the value of | log FC |. (B) and (C) GO, KEGG enrichment analysis of the DEGs. (D) GSEA‐KEGG analysis of DEGs. DEGs, differential expressed genes; FC, fold change; GO, Gene Ontology; GSEA, Gene Set Enrichment Analysis. KEGG, Kyoto Encyclopedia of Genes and Genomes.

To better assess the immune cell status of APDS, we used the “CIBERSORTx” website to estimate the proportion of immune cell subpopulations in the patient and his parents. As shown in Figure 3, eight differentially altered immune cells were detected between the son and the parents. The proportion of B cells memory, T cells CD4 memory resting and dendritic cells activated were reduced, whereas B cells naïve, T cells CD8, NK cells resting, monocytes, and macrophages M2 were increased in the child.

**FIGURE 3:**
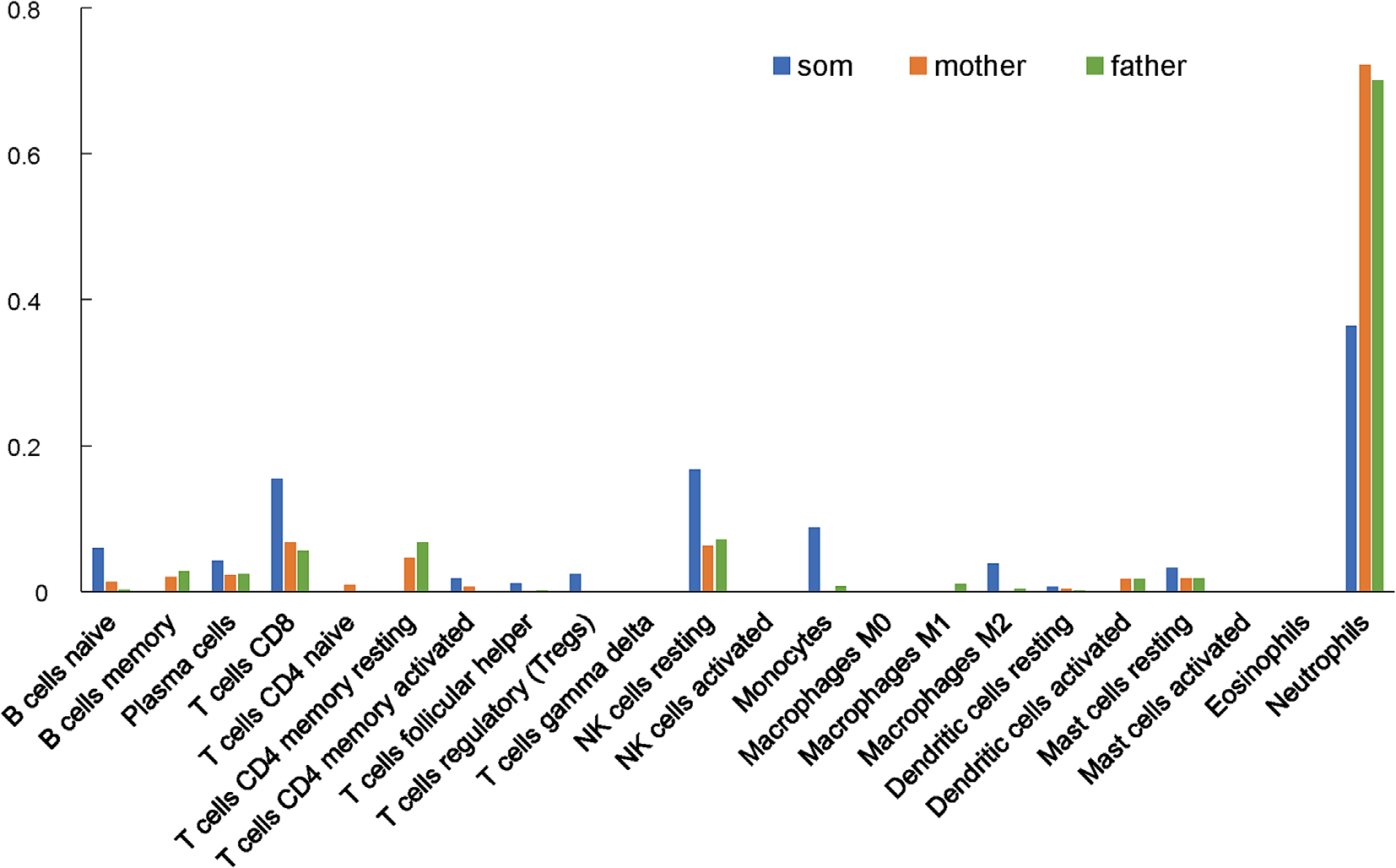
Estimated proportion of the immune cell subpopulations in the child with activated phosphoinositide 3‐kinase delta syndrome (APDS) and his parents.

Next, we explored the hub gene expressions and immune cell correlations. A total of 518 common DEGs were detected between the patient and his parents (323 upregulated, 131 downregulated) (Figure 4A and B). The top 10 hub genes were screened: granzyme B (*GZMB*), Fc fragment of IgG receptor III (*FCGR3B*), granzyme A (*GZMA*), killer cell lectin‐like receptor K1 (*KLRK1*), programmed cell death 1 (*PDCD1*), CD274 molecule (*CD274*), TNF receptor superfamily member 4 (*TNFRSF4*), T cell immunoglobulin and ITIM domain (*TIGIT*), CD244 molecule (*CD244*), and CD160 molecule (*CD160*) (Figure 4C). Only CD274 and FCGR3B were downregulated in the patient. We then analyzed the association of hub genes with differentially altered immune cells. As shown in Figure 4D, all hub genes showed moderate to strong relatedness with immune cell proportions.

**FIGURE 4:**
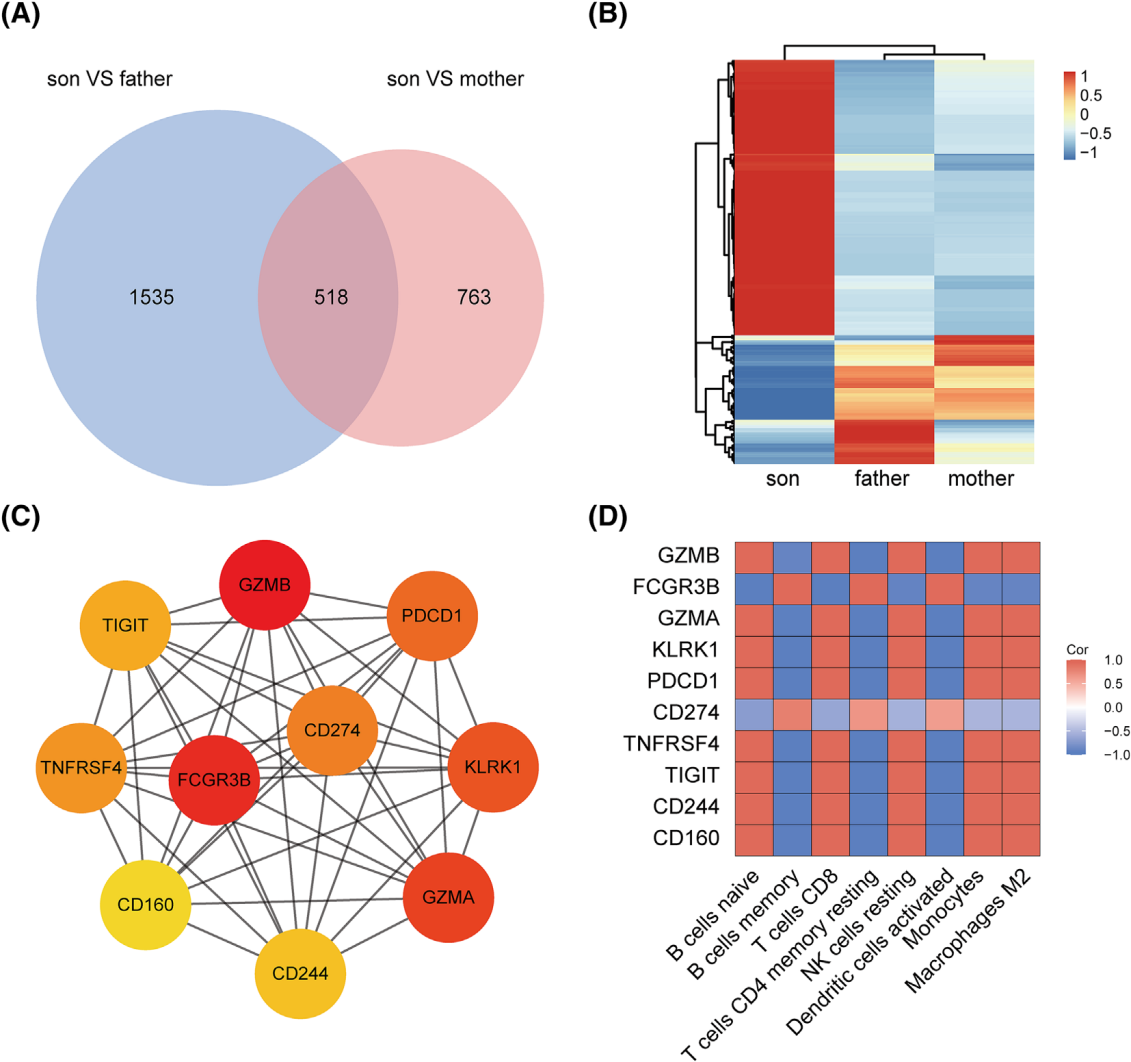
Correlation analyses of hub genes and immune cells. (A) Venn diagrams indicating the 518 common differential expressed genes (DEGs) shared by the child and his parents. (B) Heatmaps showing the common DEGs. The gradation of color represents the value of | log FC |. (C) CytoHubba was used to construct the top 10 hub genes. (D) A correlation heat map was used to show the correlation between the hub genes and differentially altered immune cells. Red depicts a positive correlation, whereas blue indicates a negative correlation. The color scale bar (0–1) corresponds to weak and strong correlations.

Here, we attempted to use bulk RNA sequencing to assess immune cells status in a child with APDS. The GO function enrichment analysis showed that DEGs were primarily involved in both innate immune response and adaptive immune response‐related functions. The DEG signaling pathways were significantly enriched in virus infection as well as the PI3K/Akt, MAPK, natural killer cell‐mediated cytotoxicity, and NOD‐like receptor signaling pathways. Activation of the PI3K/Akt signaling pathway is now among the well‐established immunological characteristics of APDS, which plays a critical role in lymphocyte survival and proliferation.^2^, ^3^ The MAPK signaling pathway is also an important regulator that controls various cellular processes, including differentiation, proliferation, and apoptosis.^4^ Patients with mutations related to the RAS‐MAPK pathway had high percentages of immature B cells, similar to patients with APDS.^5^ NOD‐like receptor signaling pathway plays vital roles in the inflammatory response, and a Gene Set Enrichment Analysis (GSEA) showed that this pathway was significantly suppressed in the child. Consistent with previous reports, PI3K overactivation substantially decreased in B cells memory and T cells CD4 memory resting counts but increased B cells naïve, T cells CD8, and NK cells resting counts.^2^, ^3^ The percentages and functions of DCs and macrophages had not yet been reported. Some in vitro studies suggested that PI3K/AKT signaling activation could downregulate inflammatory responses in DCs and mature macrophages, and that the immunosuppressive state accelerates monocytes transformation to M2 macrophages.^6^, ^7^ These provided an explanation for our results: activated dendritic cells counts decreased, and macrophages M2 counts increased. Together, these results indicate that the immune cells in patients with APDS may be in a state of immunosuppression.

Overall, deconvolution of bulk RNA sequencing to assess immune cells status can provide further insight into the alterations in immunological features, immune cell functions, and signaling pathways caused by continuous PI3K pathway activation. At present, the speculation based on mutations cannot reflect the immune status of specific children. The tests of immune function, including single‐cell sequencing, high‐throughput flow cytometry, and specific immune function tests for in vitro stimulation, cannot be carried out in clinic at low cost and quickly. We attempted to detect mixed samples of peripheral blood and analyzed the immune profile of the patient through bioinformatics analysis, which can provide reference for clinical practice. This study provides a simple, efficient, and low‐cost method for the detection of the immune system in children with immunodeficiency. With the accumulation of data, it will provide support for clinical diagnosis and treatment.

## AUTHOR CONTRIBUTIONS

**Xia Huang**: Drafting, analysis and review of literature. **Haiyan Gu**: Study design; financial support. All authors read and approved the final manuscript.

## CONFLICT OF INTEREST STATEMENT

The authors declare no conflicts of interest.

## ETHICS STATEMENT

The study was approved by the research ethics committee of the Children's Hospital of Nanjing Medical University (Approval number: 202208160‐1) and the parents of the child provided written informed consent prior to inclusion in the study.

## Supporting information


**Data S1.** Supporting Information.

## Data Availability

The data that support the findings of this study are available from the corresponding author upon reasonable request.
