# Supplementary material for: Deconvolution of bulk RNA sequencing in activated phosphoinositide 3‐kinase δ syndrome
Source: Clin Respir J. 2023 Sep 27;17(11):1190–5. doi: 10.1111/crj.13702 (PMC10632076; doi:10.1111/crj.13702)
Supplement: Supplementary file 1 — Data S1. Supporting Information. [file CRJ-17-1190-s001.doc]

**Deconvolution of bulk RNA sequencing to assess immune cells status in Activated PI3Kδ delta Syndrome**

Xia Huang; Haiyan Gu

**MATERIALS AND METHODS**

**Isolate peripheral leukocytes**

The study was approved by the research ethics committee of the Children's Hospital of Nanjing Medical University (Approval number: 202208160-1) and the parents of the child provided written informed consent prior to inclusion in the study. Whole blood was obtained from a patient with Activated phosphoinositide 3-kinase delta syndrome (APDS) and his parents. Leukocytes were isolated from blood specimens by using the RBC lysis method (Beyotime C3702) according to the manufacturer's procedure and immediately stored with 1 mL of TRIzol reagent (Thermofisher, 15596018), respectively, in a -80°C freezer^1^.

**RNA extraction and sequencing analyses**

Total RNA was extracted using Trizol reagent (Thermofisher, 15596018) following the manufacturer's procedure. The RNA libraries were sequenced on the illumina NovaseqTM 6000 platform by LC Bio Technology CO.,Ltd (Hangzhou, China).

**Differentially expressed genes (DEGs) and enrichment analyses**

Differentially expressed genes (DEGs) analysis was performed by edgeR between two samples^2^. The genes with the parameter of false discovery rate (FDR) below 0.05 and |log_2_ fold change| ≥ 1 were considered differentially expressed genes. A total of 1282 differentially expressed genes (DEGs) were identified between the son and mother (815 upregulated, 467 downregulated), while 2053 DEGs were identified between the son and father (1447 upregulated, 606 downregulated). The obtained DEGs were utilized to create a heatmap that depicted the differences in genes between the two groups. Following the log2 fold change value, the DEGs were classified into up-regulated and down-regulated genes. DEGs were then subjected to enrichment analyses of Gene Ontology (GO) functions and Kyoto Encyclopedia of Genes and Genomes (KEGG) pathways^3,4^.

**Gene set enrichment analysis (GSEA)**

The Webgestalt (http://www.webgestalt.org) platform was used for GSEA. The top10 terms of KEGG analyses were exhibited. KEGG pathways with significant enrichment results were demonstrated based on NES (Net enrichment score), gene ratio, and P value. Gene sets with |NES|>1, NOM p <0.05, and FDR q <0.25 were enrichment significant.

**Evaluation of Immune Cells**

In this study, we used the “CIBERSORTx” website (https://cibersortx.stanford.edu/) to estimate the fraction of 22 types of immune cells among patient and his parents. CIBERSORTx is an analytical tool to impute gene expression profiles and provide an estimate of the abundance of member cell types in a mixed cell population using gene expression data.

**REFERENCES**

1. Heng Z, Ruan L, Gan R. Three Methods to Purify Leukocytes and RNA Quality Assessment. *Biopreservation and biobanking.* 2018;16(6):434-438.

2. Love MI, Huber W, Anders S. Moderated estimation of fold change and dispersion for RNA-seq data with DESeq2. *Genome biology.* 2014;15(12):550.

3. Ashburner M, Ball CA, Blake JA, et al. Gene ontology: tool for the unification of biology. The Gene Ontology Consortium. *Nature genetics.* 2000;25(1):25-29.

4. Kanehisa M, Furumichi M, Sato Y, Ishiguro-Watanabe M, Tanabe M. KEGG: integrating viruses and cellular organisms. *Nucleic acids research.* 2021;49(D1):D545-d551.
